# Development of Equine IgG Antivenoms against Major Snake Groups in Mozambique

**DOI:** 10.1371/journal.pntd.0004325

**Published:** 2016-01-05

**Authors:** Felipe Raimondi Guidolin, Celso Pereira Caricati, José Roberto Marcelino, Wilmar Dias da Silva

**Affiliations:** 1 Laboratório Especial Piloto de Pesquisa e Desenvolvimento de Imunobiológicos Veterinários (LEPDIV), Instituto Butantan, São Paulo, Brazil; 2 Divisão de Desenvolvimento Tecnológico e Produção, Soros Hiperimunes, Instituto Butantan, São Paulo, Brazil; 3 Laboratório de Imunoquímica, Instituto Butantan, São Paulo, Brazil; Monash University, AUSTRALIA

## Abstract

**Background:**

Snake envenoming is a significant public health problem in underdeveloped and developing countries. In sub-Saharan Africa, it is estimated that 90,000–400,000 envenomations occur each year, resulting in 3,500–32,000 deaths. Envenomings are caused by snakes from the *Viperidae* (*Bitis* spp. and *Echis* spp.) and *Elapidae* (*Naja* spp. and *Dendroaspis* spp.) families. The African continent has been suffering from a severe antivenom crisis and current antivenom production is only sufficient to treat 25% of snakebite cases. Our aim is to develop high-quality antivenoms against the main snake species found in Mozambique.

**Methods:**

Adult horses primed with the indicated venoms were divided into 5 groups (*B*. *arietans*; *B*. *nasicornis* + *B*. *rhinoceros*; *N*. *melanoleuca*; *N*. *mossambica*; *N*. *annulifera* + *D*. *polylepis* + *D*. *angusticeps*) and reimmunized two times for antivenom production. Blood was collected, and plasma was separated and subjected to antibody purification using caprylic acid. Plasmas and antivenoms were subject to titration, affinity determination, cross-recognition assays and *in vivo* venom lethality neutralization. A commercial anti-*Crotalic* antivenom was used for comparison.

**Results:**

The purified antivenoms exhibited high titers against *B*. *arietans*, *B*. *nasicornis* and *B*. *rhinoceros* (5.18 x 10^6^, 3.60 x 10^6^ and 3.50 x 10^6^ U-E/mL, respectively) and *N*. *melanoleuca*, *N*. *mossambica* and *N*. *annulifera* (7.41 x 10^6^, 3.07 x 10^6^ and 2.60 x 10^6^ U-E/mL, respectively), but lower titers against the *D*. *angusticeps* and *D*. *polylepis* (1.87 x 10^6^ and 1.67 x 10^6^ U-E/mL). All the groups, except anti-*N*. *melanoleuca*, showed significant differences from the anti-*Crotalic* antivenom (7.55 x 10^6^ U-E/mL). The affinity index of all the groups was high, ranging from 31% to 45%. Cross-recognition assays showed the recognition of proteins with similar molecular weight in the venoms and may indicate the possibility of paraspecific neutralization. The three monospecific antivenoms were able to provide *in vivo* protection.

**Conclusion:**

Our results indicate that the anti-*Bitis* and anti-*Naja* antivenoms developed would be useful for treating snakebite envenomations in Mozambique, although their effectiveness should to be increased. We propose instead the development of monospecific antivenoms, which would serve as the basis for two polyvalent antivenoms, the anti-*Bitis* and anti-*Elapidae*. Polyvalent antivenoms represent an increase in treatment quality, as they have a wider range of application and are easier to distribute and administer to snake envenoming victims.

## Author Summary

Snake envenoming has long been a serious public health problem in underdeveloped and developing countries. In sub-Saharan Africa, the number of bites can be as high as 400,000 and result in up to 32,000 deaths. As accidents involving snakes are most common among young men in rural areas, complications and amputations can mean the loss of livelihood. Specific antivenoms are the most effective treatment available, but the only African antivenom producer can only supply 25% of the continent’s demand. In this study, we have developed antivenoms against the most important snakes commonly found in Mozambique: *Bitis* spp. (puff adders), *Naja* spp. (cobras) and *Dendroaspis* spp. (mambas). The experimental antivenoms were made by immunizing horses with the specific venoms, then collecting and processing their plasma to purify the antibodies. The experimental antivenoms were compared to the commercially available anti-*Crotalic* (rattlesnake) antivenom. The antivenoms produced had high titers, showed affinity for the specific venoms, were able to cross-recognized similar venoms and provide *in vivo* protection. The data in this study indicates that the antivenoms would be effective in treating *B*. *arietans*, *B*. *nasicornis*, *N*. *melanoleuca* and *N*. *mossambica* envenomations. We propose the development of monospecific antibodies as a strategy to increase antivenom quality, and as the basis for the production of two polyspecific antivenoms, anti-*Bitis* and anti-*Elapidae*.

## Introduction

Snake envenoming prevention and treatment has been a worldwide effort in underdeveloped and developing countries in Africa, Asia and South America. In sub-Saharan Africa, it is estimated that 90,000–400,000 envenomations occur per year, resulting in 3,500–32,000 fatalities [1]. Amputations and complications caused by snake envenoming affect 3% and 5.5% of envenomation victims, respectively [2]. As accidents with snake occur mostly with young men in rural areas, they can result in the removal of these men from the workforce, causing great personal and economic financial losses. Snake envenoming is caused mainly by snakes from the *Viperidae* (*Echis* spp. and *Bitis* spp.) and *Elapidae* (*Naja* spp. and *Dendroaspis* spp.) families. *Viperidae* snakes have a venom rich in metalloproteinases (SVMP) that can cause hemorrhagic effects and coagulatory-inducing disturbances [3]. *Echis occelatu*s is responsible for most accidents [4], while *Bitis arietans* has the widest territorial distribution [5]. *Elapidae* snake venoms have a more pronounced neurotoxic action, targeting neuromuscular junctions, and accidents can evolve to respiratory failure [6]. Spitting cobra bites (*Naja* spp.) are regarded as the most medically important due to their lethality [7]. The most effective treatment against snakebite envenoming is the administration of specific antivenom. Antivenom was introduced in Africa in 1950; there were three major producers–Behringwerke A.G. (Germany), Sanofi-Pasteur (France) and the indigenous South African Institute for Medical Research (SAIMR) [8]. After the 1980’s, the European companies ceased or greatly reduced their production due to the high cost of antibody production, and SAIMR struggled financially. The present production of antivenom (200,000 ampules/year) meets less than 25% of the African continent’s demand for snakebite treatment [9]. In an effort to solve the problem, African authorities began importing antivenoms from India and Asia. These antivenoms are not specific against African snakes and this treatment has little efficacy, causing the population to be distrustful and look for alternatives, such as traditional healing routes [10]. Even with a new wave of antivenoms being researched [11, 12, 13], there is still much to be done towards fighting snakebite envenomation in sub-Saharan Africa. In this study, we concentrate on the development of antivenoms against eight snake species found in Mozambique: *Bitis arietans*, *B*. *nasicornis*, *B*. *rhinoceros*, *Naja melanoleuca*, *N*. *mossambica*, *N*. *annulifera*, *Dendroaspis angusticeps* and *D*. *polylepis*. Our results are promising, and provide the basis for polyvalent antivenoms that could prove viable for widespread use.

## Methods

### Reagents

Tris buffer (Tris HCl, 25 mM; pH 7.4), complete MMT80 (Marcol Montanide ISA 50, 2 mL; sodium chloride 0.15 M, 5 mL; Tween 80, 1 mL; lyophilized BCG, 1 mg), incomplete MMT80 (Marcol Montanide ISA 50, 2 mL; sodium chloride 0.15 M, 5 mL; Tween 80, 1 mL), solution A for SDS buffer (Tris, 6.25 mM; SDS, 6.94 mM; pH 6.8), SDS buffer for non-reducing conditions (solution A, 8.5 mL; glycerol, 1 mL; bromophenol blue 1%, 2 mL), PBS buffer (potassium chloride, 2.6 mM; monobasic potassium phosphate, 1.5 mM; sodium chloride, 76 mM; disodium phosphate, 8.2 mM; pH 7.2–7.4), AP buffer (Tris HCl, 100 mM; sodium chloride, 100 mM; magnesium chloride, 5 mM; pH 9.5), NBT solution (NBT, 50 mg; dimethylphormamide, 700 μL; H_2_O, 300 μL), BCIP solution (BCIP, 50 mg; dimethylphormamide, 1 mL), developing solution for Western blot (AP buffer, 5 mL; NBT solution, 33 μL; BCIP solution, 16.5 μL), citrate buffer (citric acid, 0.1 M; monobasic sodium phosphate, 0.2 M; pH 5.0), OPD solution (OPD, 20 mg; citric acid, 1 mL) and substrate buffer for ELISA (citrate buffer, 5 mL; OPD solution, 100 μL; H_2_O_2_ 30 volumes, 5 μL) were used. Except for the NBT/BCIP, obtained from Molecular Probes (USA), the reagents used were obtained from Sigma-Aldrich (USA).

### Quantification of proteins

The protein concentrations of the venoms and plasma/antivenoms were assessed by the bicinchoninic acid method [14] using a Pierce BCA Protein Assay Kit (Rockford, IL).

### Venoms

*B*. *arietans*, *B*. *nasicornis*, *B*. *rhinoceros*, *N*. *melanoleuca*, *N*. *mossambica*, *D*. *angusticeps*, *D*. *polylepis* and *N*. *annulifera* venoms were supplied by Venom Supplies Pty Ltd (59 Murray Street, Tanunda, Australia) and stored at Laboratório de Venenos, Instituto Butantan. Each venom batch was made from sample mixtures of several snake specimens and lyophilized.

### Animals

Adult horses (400–450 kg) were used to produce the anti-venoms, and they were divided into 5 groups: anti-*B*. *arietans*, n = 12; anti-*B nasicornis* + *B*. *rhinoceros*, n = 12; anti-*N*. *melanoleuca*, n = 12; anti-*N*. *mossambica*, n = 6; anti-*D*. *angusticeps* + *D*. *polylepis* + *N*. *annulifera*, n = 9. The animals were primed (4 injections, 15 days apart) and maintained in a special animal house at the São Joaquim Farm, Instituto Butantan, São Paulo, Brazil. Before immunization, the animals were vaccinated against common equine infectious diseases.

### Ethical clearance

All animals used in this study were maintained and treated under strict ethical conditions in accordance with the “International Animal Welfare recommendations” [15] and the “Committee Members, International Society on Toxinology” [16]. This project was approved by the Ethics Committee of Animal Usage in Research (Protocol No: 1137/13) of the Instituto Butantan.

### Antigen preparation, immunization schedule and antivenoms

The horses received the following mixtures: anti-*B*. *arietans* (n = 12), 3.5 mg/animal of crude *B*. *arietans* venom; anti-*B*. *nasicornis* + *B*. *rhinoceros* (n = 12), 3.5 mg/animal of crude *B*. *nasicornis* and *B*. *rhinoceros* venom mixture (1:1); anti-*N*. *melanoleuca* (n = 12), 3.5 mg/animal of crude *N*. *melanoleuca* venom; anti-*N*. *mossambica* (n = 6), 3.5 mg/animal of crude *N*. *mossambica* venom; anti-*D*. *angusticeps* + *D*. *polylepis* + *N*. *annulifera* (n = 9), 3.5 mg/animal of crude *D*. *angusticeps*, *D*. *polylepis* and *N*. *annulifera* venom mixture (1:1:1). The subcutaneous injections were performed 15 days apart at four different points in the dorsal region of each animal. The animals were primed (4 inoculations in 6 mL of complete or incomplete MMT80 adjuvant), and later re-immunized for this experiment (2 inoculations in 6 mL of PBS). Fifteen days after each inoculation post-priming, blood was collected (8 mL/animal) in vials with anticoagulant solution (heparin by venipuncture of the jugular vein). The plasma was separated by centrifugation (1,500 rpm for 15 min at 4°C) and stored at -20°C. Horse blood was collected and the plasma was separated as described above. Four equine plasma samples (Batch No: #143, #158, #223 and #356) from horses immunized with *C*. *d*. *terrificus* venom, according to WHO guidelines [17], were used to produce anti-*Crotalic* serum. These samples were provided by “Divisão de Desenvolvimento Tecnológico e Produção–Seção de Processamento de Plasmas Hiperimunes, Instituto Butantan”, and they were used as a standard for comparison. The obtained plasma samples were pooled within their respective groups and immunoglobulins were purified using caprylic acid.

### Antibody purification with caprylic acid

Equine antibodies were purified from plasma (2^nd^ immunization samples) following the procedure described by Dos Santos et al. [18]. The plasma samples were pooled within their respective groups, heated at 56°C for 15 min to achieve complement inactivation and centrifuged at 900 g for 10 min. The pH of the samples was adjusted to 5.0 through the addition of 0.1 N acetic acid. Caprylic acid was added slowly with vigorous shaking to a final concentration of 8.7%, and then the samples were left to shake for 30 min at room-temperature. After a second centrifugation step (11,000 g for 15 min), the supernatants were collected, and their pH were adjusted to between 7.0 and 7.5 through the addition of 0.1 N NaOH. The samples were filtered through a Sterile Millex 0.45 μm (Milliford Corporation, Billeric, MA). Dialysis was performed against PBS buffer using a Centricon 50 kDa Centrifugal Device (Milliford Corporation, Billeric, MA) 4,000 g for 10 min, three times. The samples were then diluted to 30 mg/mL and stored at -20°C.

### Quantification of the antivenom antibodies

Polystyrene, high-affinity ELISA plates (96 wells) were coated with 1.0 μg/well of crude *B*. *arietans*, *B*. *nasicornis*, *B*. *rhinoceros*, *N*. *melanoleuca*, *N*. *mossambica*, *D*. *angusticeps*, *D*. *polylepis* or *N*. *annulifera* venoms in 100 μL of PBS buffer and kept overnight at 4°C. In some assays, the plates were coated with 1.0 μg/well of crude *C*. *d*. *terrificus* venom for standard antivenom quantification. The plates were blocked for 2 h at 37°C with 200 μL/well of PBS plus 5% BSA. The plates were washed with 200 μL/well of PBS. Serial dilutions of horse plasma (1:4,000 to 1:512,000) or IgG antivenoms (1:2,000 to 1,024,000) in PBS plus 0.1% BSA were prepared, and 100 μL/well of each dilution was added to their respective antigens. The plates were then incubated at 37°C for 1 h, and washed three times with the wash buffer (PBS plus 0.1% BSA and 0.05% Tween-20, 200 μL/well). Rabbit peroxidase-conjugated anti-horse IgG (whole molecule) (Sigma Aldrich, St. Louis, MO) diluted (1:20,000) in PBS plus 0.1% BSA (100 μL/well) was added to the plates. The plates were incubated for 1 h at 37°C. After three washes with the wash buffer, 50 μL/well of the substrate buffer was added, and the plates were incubated at room temperature for 15 min. The reaction was terminated by the addition of 50 μL/well of 4 N sulfuric acid. The absorbance at 492 nm was recorded using an ELISA plate reader (Labsystems Multiskan Ex, Thermo Fisher Scientific Inc., Walthan, MA). IgG from horses collected before immunization was used as a negative control (fixed dilution of 1:2,000). The antivenom dilution with an optical density of 0.2 was used to calculate the U-ELISA per milliliter of the undiluted antivenom solution. One U-ELISA was defined as the smallest dilution of antibody that presented an OD of 0.2 under the conditions used in the ELISA assay, as described previously [19]. The value was then multiplied by 10 to convert it to milliliters.

### Antivenom affinity measurements

The affinities of the horse plasmas (2^nd^ immunization samples) or IgG antivenoms were measured using ELISA, following the methodology described above, with the inclusion of a potassium thiocyanate (KSCN) elution step [20, 21, 22]. After the serum incubation step, dilutions of KSCN (0.0 to 5.0 M, in intervals of 1.00 M) in distilled H_2_O were added to the wells and incubated for 30 min at room temperature. The remaining bound antibodies were detected with rabbit peroxidase-conjugated anti-horse IgG (whole molecule) (Sigma Aldrich, St. Louis, MO) diluted (1:20,000) in PBS plus 0.1% BSA (100 μL/well). After three washes with the wash buffer, 50 μL/well of substrate buffer was added, and the plates were incubated at room temperature for 15 min. The reaction was terminated with 50 μL/well of 4 N sulfuric acid. The absorbance at 492 nm was recorded using an ELISA plate reader (Labsystems Multiskan Ex, Thermo Fisher Scientific Inc., Waltham, MA). The results are expressed as follows: affinity index (AI) = % bound antibodies at KSCN 5 M.

### SDS-PAGE and western blot cross-recognition essay

Western blot analysis was carried out according to the method previously described by Towbin et al. [23]. Crude *B*. *arietans*, *B*. *nasicornis*, *B*. *rhinoceros*, *N*. *melanoleuca*, *N*. *mossambica*, *D*. *angusticeps*, *D*. *polylepis* and *N*. *annulifera* venoms (10 μg) were treated with non-reducing SDS-PAGE sample buffer and resolved in 12.5% polyacrylamide gel. Gels were either submitted to silver staining or electroblotted onto nitrocellulose membranes, according the method described by Laemmli [24]. These membranes were blocked with PBS buffer containing 5% BSA at 37°C for 2 h, washed with PBS, and treated with the antivenom diluted to 1:20,000 in PBS plus 0.1% BSA for 1 h at room temperature on a horizontal shaker. Each membrane was treated with only one antivenom. After being washed three times with PBS plus 0.05% Tween-20, the membranes were incubated with rabbit anti-horse IgG conjugated to alkaline phosphatase (whole molecule) diluted to 1:7,500 in PBS plus 0.1% BSA. Then, the membranes were incubated for 1 h at room temperature on a horizontal shaker. The membranes were washed three times with PBS plus 0.05% Tween-20 and placed in developing solution for Western blotting. The reaction was terminated by washing with distilled water.

### *In vivo* lethality neutralization essay

Male Swiss mice, 18–20 g, were used in protocols to determine the lethality (LD_50_) of the venoms and the neutralizing potency (ED_50_) of the antivenoms. For LD_50_ determination, different venom quantities were prepared in 0.85% NaCl solution and intraperitoneally injected (500 μL) in the mice. Four mice were used per venom dose. Deaths were recorded after 48 h, and LD_50_ was estimated by probits analysis [25]. For ED_50_ determination, a fixed amount of 3 LD_50_ of snake venom and various dilutions of the respective purified antivenoms (1:5, 1:10, 1:20, and 1:40) were incubated for 30 min at 37°C. Venom samples incubated only with 0.85% NaCl solution were used as controls. After incubation, 500 μL aliquots of the mixtures were intraperitoneally injected in the mice. Four mice were used per dilution. The death/survival ratio was recorded at 3 h, 24 h and 48 h after the injection. ED_50_ was estimated by probits analysis. Data is expressed as Specific Activity (μg of venom neutralized by 1mg of antibodies).

### Statistical analysis

The data was analyzed using one-way ANOVA, followed by the Dunnett’s Multiple Comparison Test (standard antivenom as comparison), or two-way ANOVA, followed by the Bonferroni Post-Test (standard antivenom as comparison). Differences were considered to be significant if P < 0.05. Analysis was performed using GraphPad Prism 5 for Windows (GraphPad Software, San Diego, USA).

## Results

### Hyperimmune plasma titration

**Fig 1:**
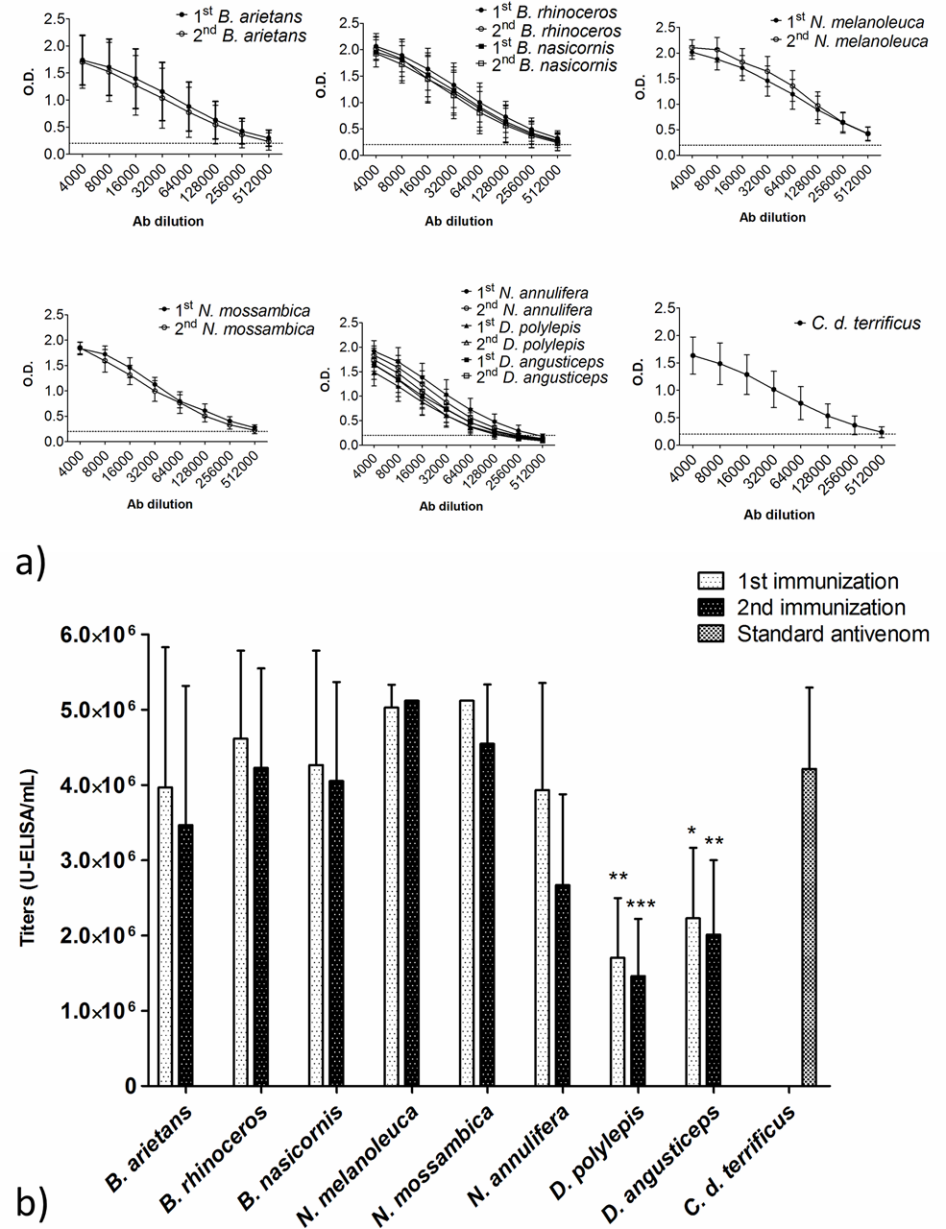
Titration from experimental plasmas and standard anti-*Crotalic* antivenom before IgG purification. (a)Titration curves and (b) titers comparison. The plates were sensitized with 1 μg of antigen/well and the plasma dilutions ranged from 1:4,000 to 1:512,000. The dotted line represents an OD of 0.2. The titers are expressed as Units-ELISA/mL x 10^6^. The medians were compared by two-way ANOVA followed by the Bonferroni Post-Test (standard antivenom as comparison), *P < 0.05, **P < 0.01, and ***P < 0.001. The data are representative of three independent experiments.

The titration of the plasmas against the respective venoms was performed using ELISA. The plates were sensitized with 1 μg/well of antigen, and the plasma dilutions ranged from 1:4,000 to 1:512,000. Titration curves from different immunizations and groups showed similar kinetics (Fig 1A). When titers were compared, differences between different immunizations were not found (Fig 1B). The groups anti-*B arietans* and anti-*B*. *nasicornis* + *B*. *rhinoceros* showed similar results, with titers ranging from 3.47 x 10^6^ to 4.62 x 10^6^ U-ELISA/mL. The anti-*N*. *mossambica* and anti-*N*. *melanoleuca* groups displayed the highest titers, 4.55 x 10^6^ to 5.12 x 10^6^ U-ELISA/mL. Significant differences were not found between those groups and the standard anti-*Crotalic* antivenom (4.21 x 10^6^ U-ELISA/mL). The group anti-*D*. *angusticeps* + *D*. *polylepis* + *N*. *annulifera* showed higher titers against *N*. *annulifera* venom (from 2.67 x 10^6^ up to 3.93 x 10^6^ U-ELISA/mL), and lower titers against the *Dendroaspis* venoms, between 1.46 x 10^6^ and 2.23 x 10^6^ U-ELISA/mL. This group was also the only one to show a statistically significant difference when compared to the standard anti-*Crotalic* antivenom.

### Hyperimmune plasma affinity determination

**Fig 2:**
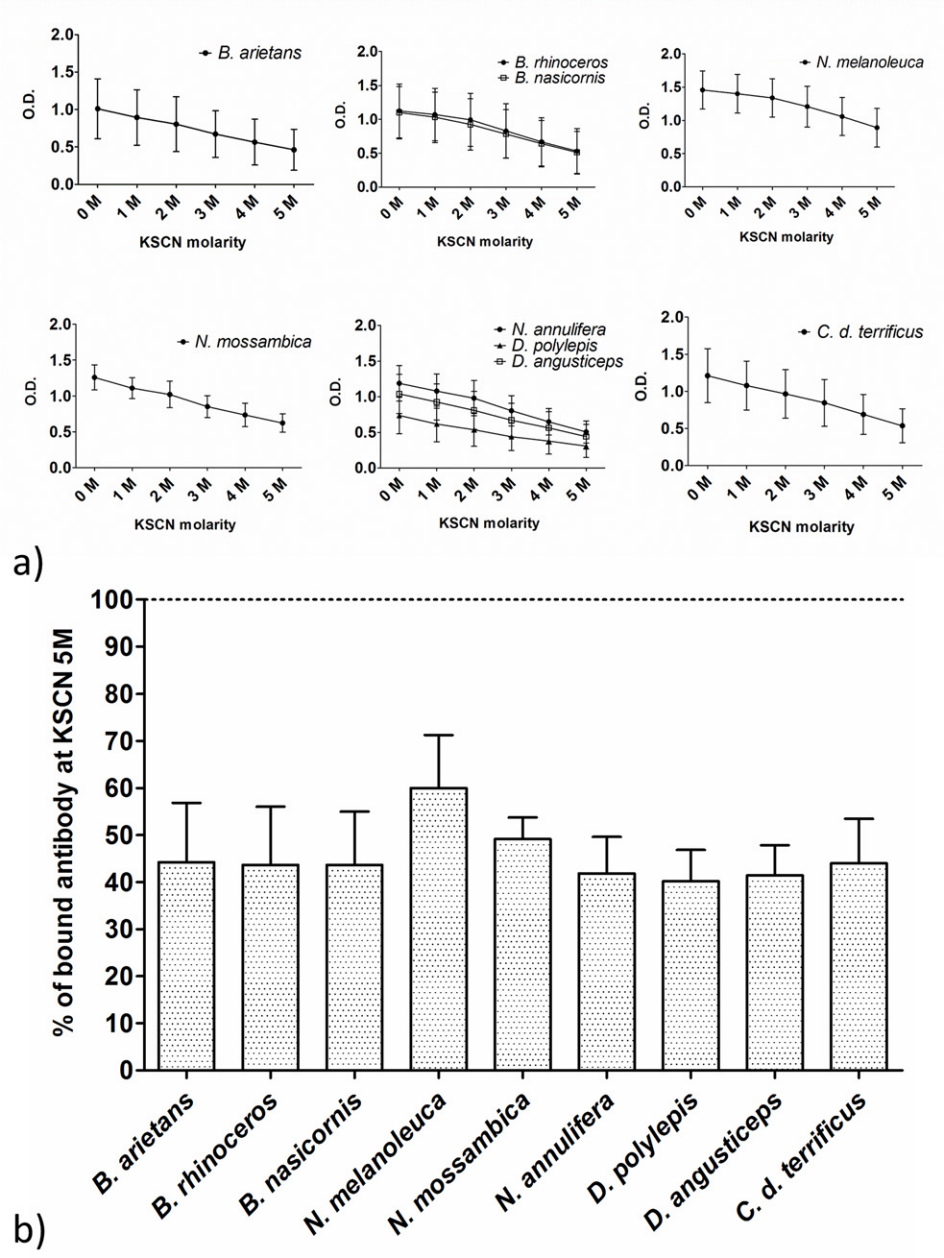
Affinity of experimental plasmas (2^nd^ immunization) and standard anti-*Crotalic* antivenom before IgG purification. (a) Affinity curves and (b) affinity index comparison. The plates were sensitized with 1 μg of antigen/well, and the plasma dilutions were fixed at 1:20,000. The KSCN dilution ranged from 0 M to 5 M in 1 M intervals. The affinities were calculated as the percentage of antibodies bound at KSCN equal to 5 M; the dotted line represents 100%. The medians were compared by one-way ANOVA followed by the Dunnett’s Multiple Comparison Test (standard antivenom as comparison), *P < 0.05. The data represent three independent experiments.

Affinity determination was performed by ELISA with the inclusion of an elution step with KSCN, a chaotropic agent, in concentrations ranging from 0 M to 5 M. Only plasma samples from the 2^nd^ immunization were used, and the dilution was fixed at 1:20,000. The affinity curves obtained for all groups presented similar kinetic behaviors (Fig 2A). The affinity index determination showed a similar percentage of bound antibodies between the experimental groups with the affinity index ranging from 40% to 49% against most of the venoms tested (Fig 2B). The highest affinity was obtained with the anti-*N*. *melanoleuca* plasma (60%). No statistical significant difference was found between the experimental groups and the standard anti-*Crotalic* antivenom (44%).

### Antibody preparation titration

**Fig 3:**
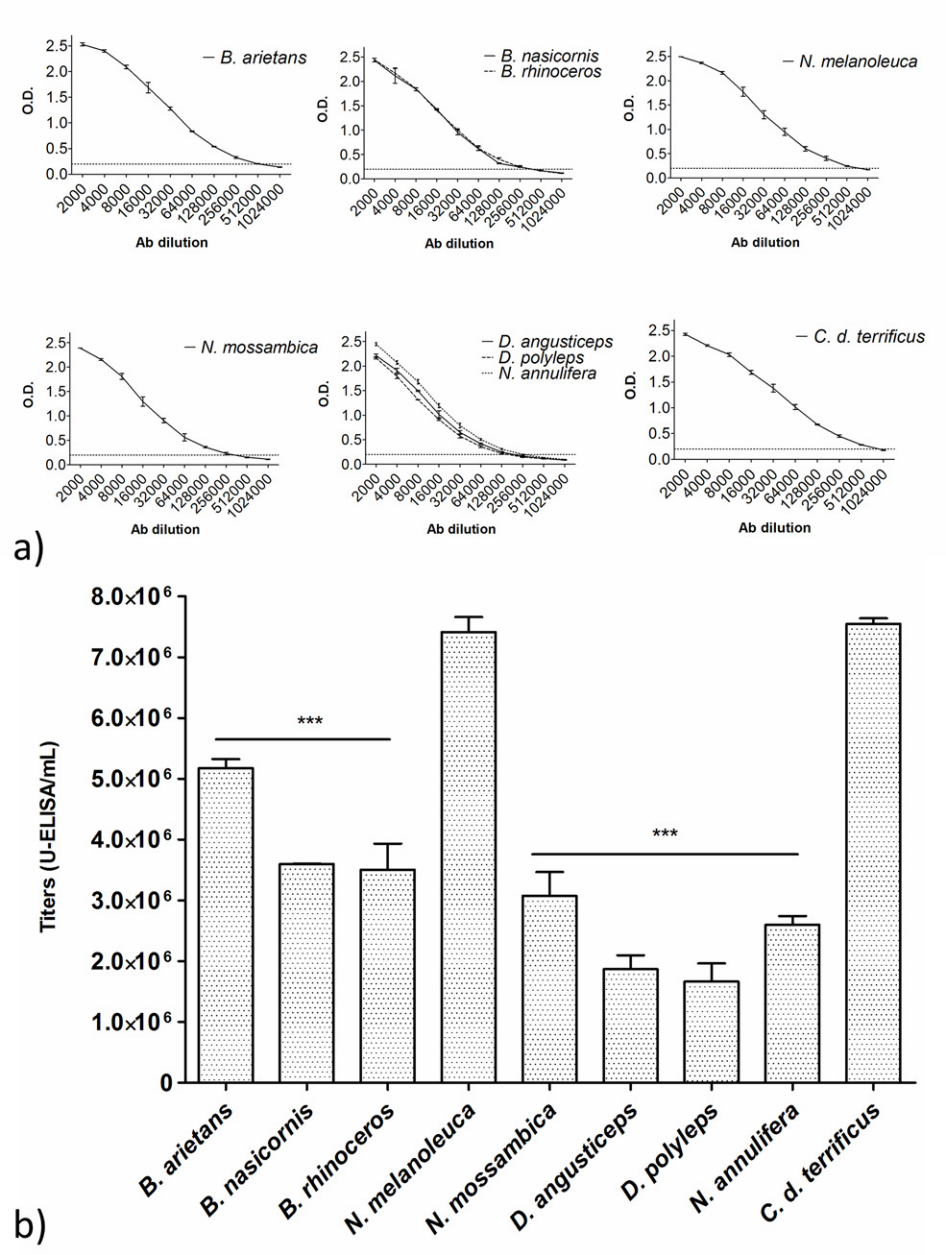
Titration from experimental and standard (anti-*Crotalic*) antivenoms after IgG purification. (a) Titration curves and (b) titer comparison. The plates were sensitized with 1 μg of antigen/well and the plasma dilutions ranged from 1:2,000 to 1:1,024,000. The dotted line represents an OD of 0.2. The titers are expressed as Units-ELISA/mL x 10^6^. Medians were compared by one-way ANOVA followed by the Dunnett’s Multiple Comparison Test (standard antivenom as comparison), ***P < 0.05. The data represent three independent experiments.

Plasmas samples (2^nd^ immunization) were used for the IgG antibody purification with caprylic acid. The titration of the resulting IgG antivenoms was performed with ELISA, as described previously, with antibody dilutions ranging from 1:2,000 to 1:1,024,000. The titration curves again displayed similar kinetics, with a more pronounced decline towards greater antibody dilutions (Fig 3A). We observed a small increase in titers after purification in the anti-*B*. *arietans* (5.18 x 10^6^ U-ELISA/mL), anti-*B*. *nasicornis* + *B*. *rhinoceros* (3.50 x 10^6^ and 3.60 x 10^6^ U-ELISA/mL, respectively) and an small decrease in titers in anti-*D*. *angusticeps* + *D*. *polylepis* + *N*. *annulifera* (1.87 x 10^6^, 1.67 x 10^6^ and 2.60 x 10^6^ U-ELISA/mL, respectively) groups. The anti-*N*. *mossambica* group had a high decrease in titers (3.07 x 10^6^ U-ELISA/mL), while the anti-*N*. *melanoleuca* group showed a big increase; it had the highest titers (7.41 x 10^6^ U-ELISA/mL). We found statistically significant differences between all the groups, with the exception of anti-*N*. *melanoleuca*, when compared to the purified standard anti-*Crotalic* antivenom (7.55 x 10^6^ U-ELISA/mL) (Fig 3B).

### Antivenom affinity determination

**Fig 4:**
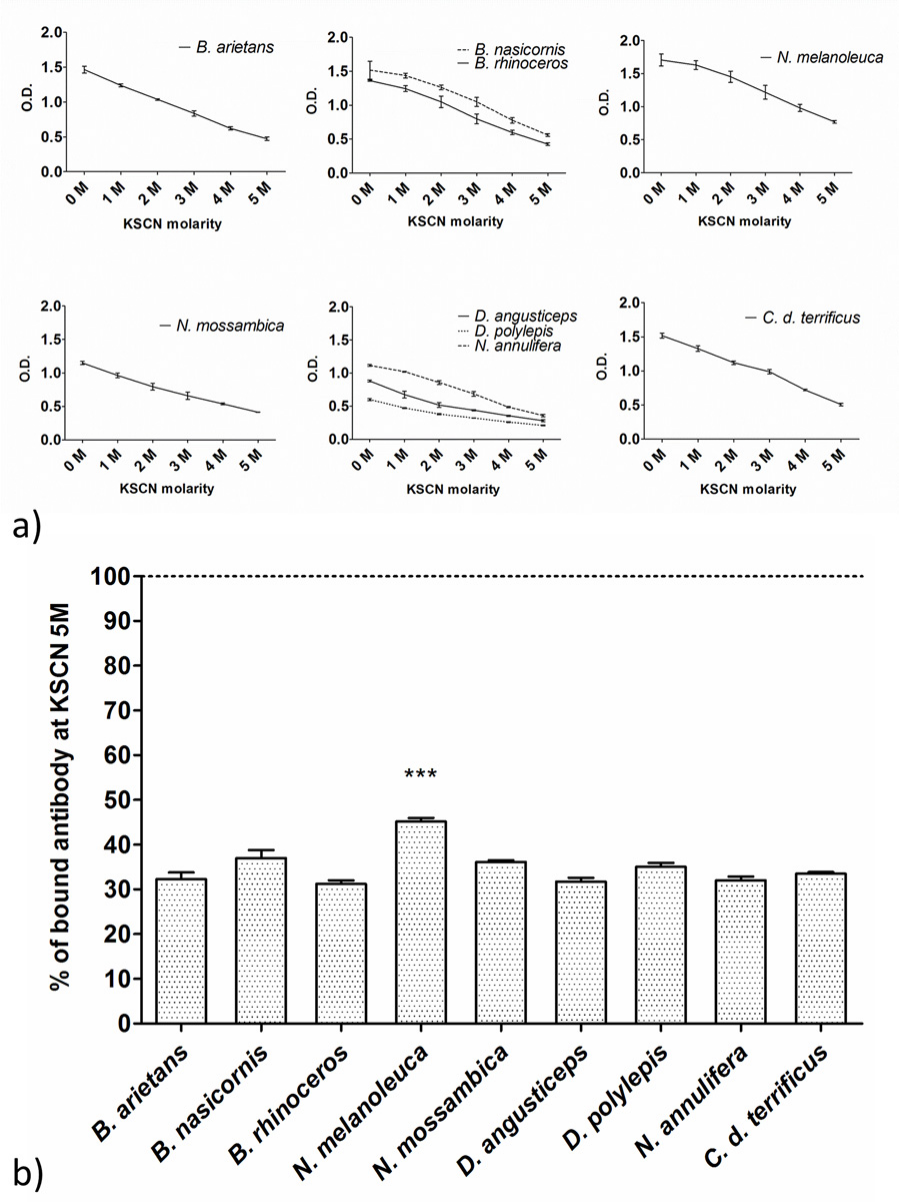
Affinity of experimental and standard (anti-*Crotalic*) antivenoms after IgG purification. (a) Affinity curves and (b) affinity index comparison. The plates were sensitized with 1 μg of antigen/well and the plasma dilutions fixed at 1:20,000. The KSCN dilution ranged from 0 M to 5 M in 1 M intervals. The affinity was calculated as the percentage of antibodies bound at KSCN equal to 5 M; the dotted line represents 100%. The medians were compared using a one-way ANOVA followed by the Dunnett’s Multiple Comparison Test (standard antivenom as comparison), ***P < 0.05. The data represent three independent experiments.

The affinity curves displayed similar kinetics both between groups and with pre- and post-purification samples (Fig 4A). The affinity indices decreased compared to the pre-purification samples, ranging between 31% and 37% against most venoms. The anti-*N*. *melanoleuca* group showed the highest results (45% bound antibodies at KSCN 5 M). This group was also the only one to show a statistically significant difference when compared to the standard anti-*Crotalic* antivenom (33%) (Fig 4B).

### Cross-recognition by western blot

**Fig 5:**
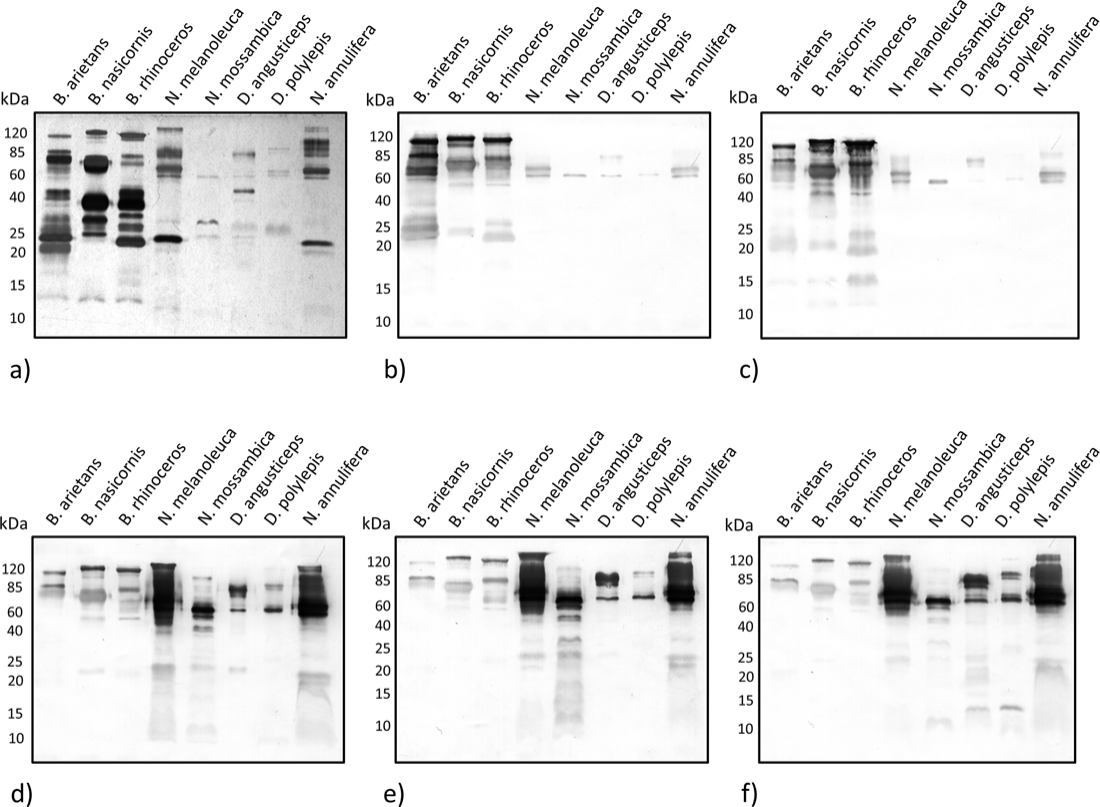
Electrophoretic profile and cross-recognition by antivenoms as assessed by western blotting (WB). (a) SDS-Page, upper gel 5%, lower gel 12.5%, stained with silver. (b) WB using anti-*B arietans* antivenom. (c) WB using anti-*B nasicornis + B*. *rhinoceros* antivenom. (d) WB using anti-*N*. *melanoleuca* antivenom. (e) WB using anti-*N*. *mossambica* antivenom. (f) WB using anti-*D*. *angusticeps + D*. *polylepis + N*. *annulifera* antivenom. In all experiments, 10 μg of crude venom was resolved under non-reducing conditions. The antibody dilution was fixed at 1:20,000.

The venoms (10 μg) were treated with non-reducing SDS-PAGE resolved in 12.5% polyacrylamide gel. Samples were either stained with silver (Fig 5A) or electroblotted onto nitrocellulose membranes for Western blot (Fig 5B–5F) recognition with the purified antibodies (2^nd^ immunization), diluted 1:20,000. The venom from *B*. *arietans*, *B*. *nasicornis* and *B*. *rhinoceros* showed a similar pattern, with protein bands close to 15 kDa, related to PLA_2_, 20–60 kDa, related to SVSPs, and 60–120 kDa, related to SVMPs [26, 27]. The *N*. *mossambica*, *N*. *melanoleuca* and *N*. *annulifera* venoms were also similar, with protein bands close to 20, 25 and 60 kDa, related to SVMPs. The *N*. *melanoleuca* and *N*. *annulifera* venoms also displayed protein bands close to 15 kDa, related to PLA_2_, and two high weight proteins bands, 85 kDa (related to SVMPs) and 120 kDa (CVF) [28]. The venom from *D*. *angusticeps* and *D*. *polylepis* showed a few protein bands, close to 25, 40, 60 and 85 kDa, related to SVMPs [29]. The anti-*B*. *arietans* (Fig 5B) and anti-*B*. *nasicornis + B*. *rhinoceros* antivenoms (Fig 5C) exhibited bands close to 20, 25, 60, 85 and 120 kDa, present with the different *Bitis* venoms, and bands close to 60 kDa with the other venoms. The anti-*B*. *nasicornis + B*. *rhinoceros* antivenom was also able to resolve bands close to 15 kDa on the *Bitis* venoms. The anti-*N*. *melanoleuca* (Fig 5D) and anti-*N*. *mossambica* antivenoms (Fig 5E) showed bands close to 20–25, 60 and 85 kDa on most venoms tested, a band close to 10 kDa on the *N*. *melanoleuca* and *N*. *mossambica* venoms and a band over 120 kDa on the *N*. *melanoleuca* and *N*. *annulifera* venoms. The anti-*D*. *angusticeps* + *D*. *polylepis* + *N*. *annulifera* antivenom (Fig 5F) recognized the same bands as the monospecific *Naja* antivenoms, plus a band between 10 and 15 kDa on the *Dendroaspis* venoms (BPTI-like toxins). Protein bands with 25, 60 and 85 kDa appeared on all the venoms tested and were cross-recognized by all experimental antivenoms.

### Cross-recognition titration

**Fig 6:**
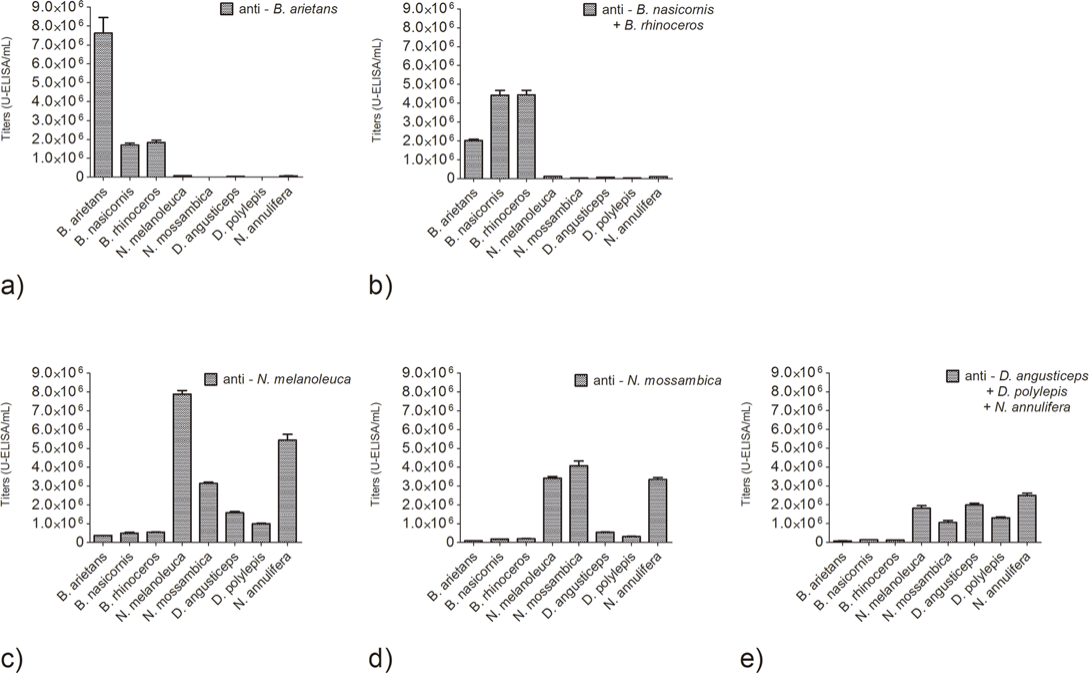
Titers obtained from experimental antivenoms in cross-recognition assays after IgG purification. (a) anti-*B*. *arietans* antivenom. (b) anti-*B*. *nasicornis + B*. *rhinoceros* antivenom. (c) anti-*N*. *melanoleuca* antivenom. (d) anti-*N*. *mossambica* antivenom. (e) anti-*D*. *angusticeps + D*. *polylepis + N*. *annulifera* antivenom. The plates were sensitized with 1 μg of antigen/well and the plasma dilutions ranged from 1:2,000 to 1:1,024,000. The titers are expressed as Units-ELISA/mL x 10^6^. The data represent two independent experiments.

Cross-recognition titration was performed using ELISA (Fig 6) against all venoms, using the purified antibodies (2^nd^ immunization) with dilutions ranging from 1:2,000 to 1:1,024,000. The anti-*B*.*arietans* (Fig 6A) and anti-*B*. *nasicornis + B*. *rhinoceros* antivenoms (Fig 6B) yielded high titers against their respective venoms (4.42 x 10^6^ U-ELISA/mL to 7.62 x 10^6^ U-ELISA/mL) and lower titers against the other *Bitis* venoms (1.70 x 10^6^ U-ELISA/mL to 2.03 x 10^6^ U-ELISA/mL). The titration was negligible against the other venoms tested. The anti-*N*. *melanoleuca* antivenom (Fig 6C) again showed the highest titers against its respective venom (7.89 x 10^6^ U-ELISA/mL), and high titers against the other *Naja* venoms (3.14 x 10^6^ U-ELISA/mL to 5.45 x 10^6^ U-ELISA/mL). Titers against *Dendroaspis* venoms were low (< 1.60 x 10^6^ U-ELISA/mL), and the titration against the *Bitis* venom was negligible. The anti-*N*. *mossambica* antivenom (Fig 6D) yielded high titers against all three *Naja* venoms (3.35 x 10^6^ U-ELISA/mL to 4.08 x 10^6^ U-ELISA/mL), and negligible titers against the venoms from other groups. The anti-*D*. *angusticeps + D*. *polylepis + N*. *annulifera* antivenom (Fig 6E) resulted in medium titers against *Naja* (1.05 x 10^6^ U-ELISA/mL to 2.49 x 10^6^ U-ELISA/mL) and the *Dendroaspis* venoms tested (1.30 x 10^6^ U-ELISA/mL to 1.99 x 10^6^ U-ELISA/mL), and it showed negligible titers against the *Bitis* venoms.

### *In vivo* venom lethality neutralization

In vivo protection (ED_50_) was determined by the injection of 3 LD_50_ of the venom, incubate with different concentrations of the respective antivenom (1:5, 1:10, 1:20, 1:40). The three monospecific antivenoms were effective in neutralizing venom lethality (Table 1). Anti-*B*. *arietans* showed the highest specific activity, with 77 μg of venom neutralized by 1 mg of antivenom, and it was followed by anti-*N*. *mossambica* (16 μg venom/mg of antivenom) and anti-*N*. *melanoleuca* (11 μg of venom/mg of antivenom). Anti-*B*. *nasicornis + B*. *rhinoceros* provided protection against *B*. *nasicornis venom* (54 μg of venom/mg of antivenom), however it was not able to protect against *B*. *rhinoceros* lethal activity. The polyspecific anti-*D*. *angusticeps + D*. *polylepis + N*. *annulifera* was not able to neutralize the lethality of any of the three venoms, and *in vivo* protection was not achieved in this group.

**Table 1:** *In vivo* venom lethallity neutralization essay.

Venom	LD_50_ (μg/animal)	Total venom quantity	ED_50_	Specific Activity (μg venom/mg antivenom)
***B*. *arietans***	50 μg	900 μg	389 μL	**77**
***B*. *nasicornis***	30 μg	540 μg	333 μL	**54**
***B*.*rhinoceros***	60 μg	1080 μg	ND	**ND**
***N*. *melanoleuca***	10 μg	180 μg	558 μL	**11**
***N*. *mossambica***	10 μg	180 μg	366 μL	**16**
***D*. *angusticeps***	30 μg	540 μg	ND	**ND**
***D*. *polylepis***	10 μg	180 μg	ND	**ND**
***N*. *annulifera***	80 μg	1440 μg	ND	**ND**

Animals were intraperitoneally injected (500 μL) with 3 LD_50_ of venom incubated (30 min at 37°C) with different dilutions of the respective antivenom (1:5, 1:10, 1:20, 1:40). Venom samples incubated with 0.85% NaCl were used as control. 4 animals were used per group. Antivenoms protein concentration was 30 mg/mL. Specific activity is expressed as μg of venom neutralized by 1 mg of antibody. ND = non-determined.

## Discussion

Polyvalent antivenoms often use multi-specific venom mixtures for immunization [8, 11, 12, 30]. Snake venoms are a very complex mixture of components with different immunogenic properties. This means that a few, or even a single more immunogenic component, can skew the immune response towards themselves, while the less immunogenic antigens are ignored by the immune system. Using monospecific antigenic mixtures, we can customize the immunization protocols to obtain the optimal response against each venom. By pooling the antivenoms afterwards, we are able to more accurately calculate the quantity of each neutralizing antibody in the final mixture. By increasing the specific activity of the antivenom, lower doses are required for treatment, thus reducing the risk of emerging undesirable reactions.

The immunization process should be oriented towards the development of high-affinity antibodies and long-lived memory B cells. The inoculation of low antigen dosages, combined with multiple inoculation sites and longer immunization intervals, will lead to clonal expansion of only the high affinity lymphocytes (for a review, consult McHeyzer-Willians et al. [31]). This strategy had already been successfully tested by our group in the context of antivenom production [22]. In this study, we were able to obtain high-quality antibodies in a short time, and little to no difference was observed between our experimental antibodies and the commercial anti-*Crotalic* antivenom produced by Instituto Butantan. *In vivo* protection was achieved with the three monospecific antivenoms, but not with the two polyspecific antivenoms.

The anti-*B*. *arietans* and anti-*B*. *nasicornis* + *B*. *rhinoceros* antivenoms yielded high titers and had high affinity scores. Western blot also revealed a very similar recognition pattern for both antivenoms. Despite these venoms having a very similar composition, the antivenoms showed little paraspecific activity, demonstrated by the drop in titers in the cross-recognition quantification assay. The anti-*B arietans* antivenom was capable of neutralizing its respective venom lethal activity, however the anti-*B*. *nasicornis* + *B*. *rhinoceros* was only able to provide protection against *B*. *nasicornis* venom. It has been shown that antivenoms containing anti-*B*. *arietans* and anti-*B*. *gabonica* antibodies were not able to neutralize the lethal activity of *B*. *nasicornis*, although they effectively neutralized *B*. *rhinoceros* venom [12]. To ensure protection against *Bitis* envenomation, we suggest the development of monospecific *B*. *arietans*, *B*. *nasicornis* and *B*. *rhinoceros* antivenoms, which would be pooled afterwards in a single anti-*Bitis* antivenom.

The anti-*N*. *melanoleuca* and anti-*N*. *mossambica* antivenoms yielded high titers and affinity. The antivenoms were also effective in providing *in vivo* protection against their respective antivenoms. The protein bands as observed by Western blot revealed a very similar recognition profile from both antivenoms, and the cross-recognition titration demonstrated paraspecific activity. Although this suggests that cross-neutralization is a possibility, it has been shown that *N*. *melanoleuca* antivenom alone is unable to neutralize the lethal activity of *N*. *mossambica* [32]. *N*. *melanoleuca* venom has mostly neurotoxic components, while *N*. *mossambica* venom also possesses important cytotoxic components. Both anti-*Naja* antivenoms should be considered when designing polyspecific antivenoms.

The anti-*D*. *angusticeps* + *D*. *polyleps* + *N*. *annulifera* antivenom showed poor results. Although titration against *N*. *annulifera* and other *Naja* venoms was satisfactory, titers against the *Dendroaspis* venom were very low. The recognition of venom proteins by Western blot also revealed a pattern very similar to the two monospecific anti-*Naja* antivenoms. Considering the profile observed in the Western blot, the difference in titers, and the presence of pro-inflammatory components in *Naja* venom [33], we concluded that the *N*. *annulifera* venom acted as the dominant antigen, directing the immune response to it. *In vivo* lethality neutralization was not determined, as the antivenom was ineffective in providing protection against any of three venoms tested. To obtain proper protection against *Dendroaspis*, we suggest the removal of *N*. *annulifera* and the production of monospecific anti-*D*. *angusticeps* and anti-*D*. *polylepis* antivenoms.

Our data supports the production of two antivenoms for use in Mozambique: an anti-*Bitis* composed of a mixture of monospecific anti-*B*. *arietans*, anti-*B*. *nasicorni*s and anti-*B*. *rhinoceros* antivenoms, and an anti-*Elapidae*, composed of a mixture of monospecific anti-*N*. *melanoleuca*, anti-*N*. *mossambica*, anti-*D*. *angusticeps* and anti-*D*. *polylepis* antivenoms. The antivenoms proposed here could be supplied in parallel, and differential diagnosis would ensure the correct choice of treatment (*Viperidae* envenomation is characterized by hemorrhage and coagulatory disturbances [3], while *Elapidae* venom has neurotoxic properties [7]). Lyophilization of the product would be required to increase shelf life and facilitate storage and distribution.

### Conclusion

The antivenoms produced in this study, specific against medically important African snakes, showed high titers, affinity, ability to cross-recognize venoms in *in vitro* essays and were capable of *in vivo* protection. The proposed polyvalent anti-*Bitis* and anti-*Elapidae* antivenoms would be effective in the treatment of snake envenoming in Mozambique and could be further developed for continental use. The availability and distribution of these antivenoms to the population in the rural areas would represent an important step in treating snake envenomation in Africa.
